# Employment relationships of Chinese expatriates: A multi-foci perspective of psychological contract

**DOI:** 10.3389/fpsyg.2023.945292

**Published:** 2023-02-06

**Authors:** Xiao Wang, Siming Wang, Mengmei Zhu

**Affiliations:** ^1^School of Business, Chongqing City Management College, Chongqing, China; ^2^School of Management, University of Glasgow, Glasgow, United Kingdom; ^3^Southampton Business School, University of Southampton, Southampton, United Kingdom; ^4^Guangzhou Academy of Fine Arts, Guangzhou, China

**Keywords:** expatriates, psychological contract, human resource management, qualitative methods, Multinational corporations

## Abstract

**Introduction:**

Research into expatriation has made a great contribution to the understanding of issues surrounding international human resource management. However, academic discussion around the subject of expatriate management remains Western-centred, neglecting the use of expatriate staffing in multinational corporations (MNCs) from Eastern countries. By adopting a multi-foci perspective of the psychological contract, the overall objective of this research is to explore the content of Chinese expatriates’ psychological contracts.

**Methods:**

This paper draws on the findings of an organisational case study and is based on semi-structured interviews with 14 expatriates.

**Results:**

The findings provide evidence that individuals have multiple simultaneous psychological contracts, each with a different focus. The contracts held by the Chinese expatriates in this sample contain predominately balanced contract beliefs, which contrast sharply to what the other authors find to be salient beliefs (e.g., transactional contract beliefs) for expatriates based on Western samples. Importantly, the most frequently listed exchange partners by the pre-departure expatriates were line managers and department managers in headquarters; individuals appreciate the respective role of each party in shaping their aspects of work conditions whilst acknowledging the simultaneous existence of such influences.

**Discussion:**

This paper has implications for expatriate management in the following ways. First, managers are encouraged to appreciate the role of multiple parties in shaping expatriates’ psychological contracts. This helps to enhance management’s understanding on the motives and demands of those expatriates. Second, policies of support and contact would aid feelings of integration. Finally, more attention should be paid to planning expatriate career prospects.

## 1. Introduction

A key theme in the field of international human resource management (IRHM) concerns expatriation; it involves the relocation of expatriates to a subsidiary located in a foreign environment for a pre-determined time (often more than 1 year in one term) to achieve specific organizational objectives before being repatriated back to their home country (Shaffer et al., 2012; Kraimer et al., 2016). The implications of this activity are twofold. From the organization’s perspective, international assignments are facilitated for a varied range of strategic purposes such as fostering innovative capabilities (Jimenéz-Jimenéz et al., 2014), transferring corporate philosophy (Scullion and Paauwe, 2005), and controlling and aligning the operations of subsidiaries with those of headquarters (Paik and Sohn, 2004). It becomes a recognized prerequisite step, in the employees’ view, to advance their career prospects (Kraimer et al., 2016).

The recent literature on expatriates and their role within MNCs has focused on two key themes (Wang, 2019). The first theme examines the key issues of those HRM practices in this field where selection and training are essential means and expatriate performance appears to be the goal (Dabic et al., 2015). More specifically, research, respectively, discusses individual-level traits (e.g., cultural intelligence) and process design for selection and training programs that positively influence expatriate adjustment and task performance (McEvoy and Buller, 2013; Zhang et al., 2020). Suggestions are provided for selecting assignees (Zhang et al., 2020), conducting cross-cultural trainings (Mohd Yusuf et al., 2021), and designing repatriation programs (Behrendt and Bader, 2019). The second theme focuses on expatriates’ experience of adjustment (Dabic et al., 2015; Harari et al., 2018) and the implication of international assignment on expatriates’ career prospect and well-being (e.g., Kanstrén and Suutari, 2021; Biswas et al., 2022). Therefore, existing studies have considered international assignments from either an organization or employee perspective, but there is a scarce of literature that examines both themes concurrently and the interplay between employee and employer.

However, there are a handful of studies that have recognized the construct of the psychological contract as a valuable framework in examining the relationships between expatriates and organizations (Perera et al., 2018; Ruiter et al., 2018; Koveshnikov et al., 2022). The notion of the psychological contract is a micro concept of employment relationship using social exchange as theoretical basis. It has been put forward as a framework for understanding employee-employer exchange relationship and essentially is concerned with employees’ perceptions of the other party’s obligations (Coyle-Shapiro and Parzefall, 2008).

While the psychological contract lens has been relatively less deployed in the field of exptriation, recent attempts have begun to address this lacuna by focusing on the role of psychological contract perceptions in expatriates’ outcomes including adjustment (Chen and Chiu, 2009; Chen, 2010; Lee et al., 2014), organizational commitment, turnover intention (Guzzo et al., 1994; Chi and Chen, 2007; Perera et al., 2018) and intrinsic career success (Ruiter et al., 2018). In addition, studies also look at the notion of a shift of expatriates’ psychological contracts from relational to transactional (Pate and Scullion, 2010), as well as the role of national culture in the perception of a psychological contract breach (Aldossari and Robertson, 2016). Furthermore, several studies have developed conceptual work incorporating psychological contracts to explain aspects of the international assignment cycle (Yan et al., 2002; Halsberger and Brewster, 2009).

In the context of international assignment, more recent studies begin to explore the mediating role of the psychological contract (Haak-Saheem et al., 2021; Koveshnikov et al., 2022). There are also studies examining the spillover effects of psychological contract breach on violation where expatriates have concurrent psychological contracts with the subsidiary and the headquarter (Schuster et al., 2022a,b). While contributing considerably to the field of expatriates’ psychological contracts, recent research has largely been outcome-oriented which focuses on how the evaluation of psychological contract state influences expatriate outcomes, mainly being addressed through a quantitative perspective. Consequently, the nature of expatriates’ psychological contracts remains largely unknown, where a key challenge is to harmonize expectations of both MNCs and expatriates (Pate and Scullion, 2010).

Moreover, the literature on expatriates’ psychological contracts remains Western-centered, the psychological contract of Asian expatriates have been insufficient explored. The context has particular implications for expatriate studies using the psychological contract lens. To clarify, the psychological contract is a cultural phenomenon, and the cultural profile of a society, with elements such as values, attitudes, norms, and beliefs, may influence information processing in relation to an employment relationship (Hui et al., 2004). Cognitive as well as motivational differences across cultures may also determine what could be considered important obligations in the exchange relationship (Conway and Briner, 2005). The psychological contract, then, can be interpreted and constructed differently depending on the social context where the employment relationship is embedded (Rousseau et al., 2000). In the view of the differences between Western and Eastern societies and “their possible implication for reactions to psychological contract breach and the psychological contract process in general” (Rousseau et al., 2000, p. 23), the recent research guided by a Western perspective may not accurately capture the content of Asian expatriates’ psychological contracts.

Further, the extant research on expatriates’ psychological contracts works from the viewpoint that each expatriate establishes one psychological contract with the organization (Sherman and Morley, 2016; Haak-Saheem et al., 2021; Koveshnikov et al., 2022). Accordingly, the organization as a whole is often assumed to be the other party to the psychological contract of an expatriate (Marks, 2001; Lavelle et al., 2007; Coyle-Shapiro and Parzefall, 2008). The approach of viewing an organization as a single contract maker tends to overlook the social context arising in the course of interactions between expatriates and different organizational units and/or agents (Conway and Briner, 2005; Perera et al., 2018).

This research examines the nature of Chinese expatriates’ psychological contracts by employing a qualitative method. In approaching this, this research acknowledges that it is plausible for expatriates to simultaneously hold multiple psychological contracts with parties across the wider context of MNCs. In this regard, this research attempts to capture how the presence of multiple parties influences the content of Chinese expatriates’ psychological contracts. This research focuses on traditional assigned expatriates (AEs) as the frame of reference. The expatriation process for assigned expatriates is typically initiated by the employing organization and is for business purposes. In clarifying this paper’s terminology, it adopts the definition of assigned expatriates provided by Aycan and Kanungo (1997):

Employees of business or government organizations who are sent by their organization to a related unit in a country which is different from their own, to accomplish a job or organization-related goal for a pre-designated temporary time period of usually more than six months and less than five years in one term (p. 250).

Accordingly, this paper addresses the following research questions:

What is the content of the psychological contract beliefs of Chinese expatriates?

- *Who are the other parties with which expatriates perceive to be the exchange partners?*
- *How does the presence of multiple parties influence the content of the psychological contract beliefs of expatriates?*

The contribution of this paper is fourfold. First, it contributes theoretically and empirically to the literature on expatriates’ psychological contracts, an area in which there is a dearth of empirical research. Second, the paper contributes to the understanding of the links between two bodies of literature from the psychological contract and international human resource management fields, yielding important insights into complex managerial problems. Third, by drawing on a multi-foci perspective of psychological contract, this study sheds some light on the nature of expatriates’ psychological contracts. Finally, this study increases the understanding of the contract beliefs of expatriates sent from an Eastern context, thereby enhancing the understanding of the importance of context in this area.

## 2. Literature review

### 2.1. The psychological contract

The psychological contract has emerged as an important analytical framework for examining employee-employer exchange relationships (Rousseau, 1989; Coyle-Shapiro and Parzefall, 2008), but has been relatively less deployed in informing the management of expatriates (Perera et al., 2018). Academic interests in the origin of the psychological contract can be traced back to the work of Argyris (1960), Levinson et al. (1962), and Schein (1965), in which this construct was introduced and assessed in the domain of the employment relationship (Conway and Briner, 2005). It was the work of Rousseau (1989) that re-conceptualized the notion of the psychological contract, which had directed the contemporary contract research. While there is a plethora of definitions proposed since then, divergent views have been found on whether this concept was rooted in expectations (Taylor and Tekleab, 2004; Conway and Briner, 2009), obligations (Robinson and Rousseau, 1994; Bal et al., 2010) or promises of the other party (Conway et al., 2011). This paper emphasizes the term of obligation in constituting the psychological contract, as it is preferred over other notions in possessing an obligatory quality for the other party to reciprocate (Rousseau, 1989). In clarifying the conceptualization of the psychological contract, this paper adopts the Rousseau’s (1989, p. 123) definition, “an individual’s beliefs regarding the terms and conditions of a reciprocal exchange agreement between the focal person and another party.”

The psychological contract is believed to be highly perceptual and unique. The obligations that underpin the psychological contract are firmly at individual-level, which may not be shared by organizations or other parties, implying that it is the employees’ perceived obligations, rather than the obligations in fact, constitute the psychological contract (Rousseau, 1990; Rousseau and Tijoriwala, 1998). In line with this, the number of obligations in the domain of an employee’s psychological contract can be very large (Ruiter et al., 2018). For the purpose of interpreting this large number of psychological contract obligations, different types of the psychological contract are distinguished. These can be characterized as transactional and relational (Rousseau, 1990), and later as a balanced contract (Rousseau, 2000).

The transactional contract contains items (obligations) that are tangible and economic in focus, with fixed terms and conditions (e.g., promotion and high pay in exchange for hard work: Rousseau, 2004). The relational contract differs from transactional contracts by emphasizing “open end collaborations, dynamic terms” (Coyle-shapiro and Parzefall, 2008, p. 13), and a broad scope of involvement in an individual’s work and life (Taylor and Tekleab, 2004). The balanced contract is comprised of features of both transactional and relational contracts by maintaining long-term involvement while allowing for changing contract as circumstances evolve (Ntalianis and Dyer, 2021; Wu et al., 2021).

Many studies have also examined the situations where employment relationship has been damaged and a large body of research on contract violation has developed (Zhao et al., 2007; Wu et al., 2021; Li et al., 2022). Contract violation was defined as the “failure of organizations or other parties to respond to an employee’s contribution in ways the individual believes they are obligated to do so” (Rousseau, 1989, p. 128). This mechanism is the main explanatory framework that linked employment relationship to subsequent employees’ attitudes and behaviors such as job dissatisfaction (Zhao et al., 2007), reduced trust in supervision (Lapointe et al., 2013), as well as lowered level of organizational commitment (Wu et al., 2021) among others.

### 2.2. Psychological contracts in the context of expatriation

In comparison to national contexts, the psychological contract lens has been relatively less deployed in the field of exptriation (Ruiter et al., 2018). In particular relation to the nature of expatriates’ psychological contract, existing research is of opinion that the expatriates’ psychological contracts are different from the psychological contracts of national employees (Zhang and Rienties, 2017; Ruiter et al., 2018). This is due to expatraites being exposed to additional uncertainties and risks during their adjustment overseas in comparison to their domestic peers (Zhang and Rienties, 2017). Consequently, expatriates’ psychological contract can cover general obligations under national employee-organization psychological contracts, as well as specific international assignment-related obligations (e.g., temporary living allowance; Guzzo et al., 1994; Pate and Scullion, 2010; Ruiter et al., 2018). More specifically, drawing on the findings from three organizational case in Western settings (two multinational corporations in Europe and one in North American), the study by Pate and Scullion (2010) argued that as a response to the tight organizational practice in a competitive international environment, expatriates moved their psychological contracts toward a transactional overtone. This was evident by expatriates expecting enhanced remuneration, promotion on return, negotiating better compensation package before international assignment and reducing their dependence on a single organization, which suggested an individualistic and calculating interpretation of the employment relationship. A more recent study by McNulty et al. (2013) suggested that expatriates’ psychological contracts can consist of both transactional and relational obligations. These obligations related to compensation, career development and family support during the assignment. This line of work therefore highlights the complex and transactional nature of Western expatriates’ psychological contracts.

While national cultural differences have been acknowledged in the broader psychological contract literature, this represents a underdeveloped line of enquiry in relation to expatriates’ psychological contracts. In the national context, studies have highlighted unique national characteristics such as the role of the caste system in India (Rousseau et al., 2000) and the individualism in Belgium (Du and Vantilborgh, 2020) that shape the content and violation of the psychological contract in that particular context.

However, research into expatriates’ psychological contracts have largely been based on Western expatriates (e.g., Pate and Scullion, 2010; Zhang and Rienties, 2017), with the exception of the study by Aldossari and Robertson (2016). The authors qualitatively examined the role of “wasta” (reliance on network relationship) in the Saudi Arabian managers’ perceptions of psychological contract breach. Their results showed that “wasta” implicitly shaped the HR practice and informal norms in the organization. This triggered repatriates’ perceptions of psychological contract breach, but does not comment on the character of expatrates’ employment relationship as the variation of Saudi Arabian context in comparison to Western context. This review of literature reveals a lack of consideration to the nature of expatriates’ psychological contract in a Non-Western context.

In particular relation to Chinese expatriates, the social values which are of importance to their psychological contracts could be referred to as *guanxi*. The meaning of *guanxi*, as pointed out by Lee and Dawes (2005), pertains to a state in which entities (e.g., human beings, objects, or forces) are connected. The indigenous Chinese concept of *guanxi* does not have a direct Western equivalent because of the intricacies of its definition, but it may be loosely translated as “connection” or “relationship” (Lin, 2011). In Western society, the reciprocal favor is often facilitated for pursuing cost–benefit balance and tends to be provided within a limited timeframe (Cropanzano and Mitchell, 2005). In contrast, *guanxi* has unique implications for guiding the reciprocal favor in Chinese society. For example, *guanxi* is categorized as an emotional investment, thus the pursuit of cost–benefit balance is not necessarily embedded in this type of exchange (Lin, 2011). This means that the recipient is expected to exceed what is offered to him/her when receiving a favor. Under this exchange, *guanxi* implies a long-term or even lifetime return (Lin, 2011). The psychological contract literature has established that perceived obligations have no universal meaning but are interpreted depending on the social context where the employment relationship is embedded. Therefore, if there are differences of society values between Western and Chinese context, the recent research guided by a Western perspective may not accurately capture the content of Chinese expatriates’ psychological contracts. The question remains as to how Chinese expatriates interpret the psychological contract with their employer? This paper attempts to increase the understanding of this important area.

### 2.3. Multi-foci perspective of the psychological contract

Existing research on expatriates’ psychological contracts appears to work from the viewpoint that each expatriate establishes one psychological contract with the organization (Lee et al., 2014; Sherman and Morley, 2016; Haak-Saheem et al., 2021; Koveshnikov et al., 2022). Accordingly, the organization as a whole is assumed to be the other party in the psychological contract of an expatriate (Marks, 2001; Lavelle et al., 2007). With regard to psychological contract literature, there is a scarcity of research attempting to explore what employees perceive to be the employer’s perspective of the contract (Coyle-Shapiro and Parzefall, 2008). To date, research has yet to build a theory regarding this point, and has tended to adopt an approach that examines an individual’s perceived exchange with a single “employer.” Accordingly, the party that embodies the employer’s perspective can vary. For example, employees may attribute the organization with humanlike qualities through the process of “anthropomorphization,” thus treating the organization itself as a party to the psychological contract (Coyle-Shapiro and Shore, 2007, p. 171; Lavelle et al., 2007).

The psychological contract literature also suggested that employees perceive various organizational agents differing in a hierarchy of authority to be the exchange partners in the psychological contracting process (Conway and Briner, 2005), including the employee’s immediate manager (Lewis and Taylor, 2001; Tekleab and Taylor, 2003), HR managers (Conway and Briner, 2005) and senior managers (Coyle-Shapiro and Kessler, 2000). Therefore, most prior contract research maintains that an individual holds a psychological contract with a unitary “employer” that is represented either by a unique person (immediate manager, HR unit, or senior manager) or the organization in itself (Rousseau, 2000, 2004; King and Bu, 2005; Coyle-Shapiro and Parzefall, 2008). Organizations, however, are large social groups in which the leadership, hierarchy and role differentiation are formally defined. An individual can experience role conflict as well as developing dual allegiance when interacting with two or more intra-organizational groups (Argyle, 1969). Therefore, the approach of viewing an organization as a single contract maker tends to overlook the social context arising in the course of interactions between expatriates and different organizational units and/or agents (Conway and Briner, 2005; Perera et al., 2018). To further this discussion, the following paragraph draws on social exchange literature to shed light on the interactions between individuals and organizations.

It is recognized in the social exchange literature that employees differentiate and react differently depending on the level of social exchange they perceive themselves having with multiple parties such as the organization as a whole, supervisors and peers (Reichers, 1985; Shore et al., 2004; Lavelle et al., 2007; Cropanzano and Rupp, 2008; Tekleab and Chiaburu, 2011; Alcover et al., 2017). For example, the theoretical work of Reichers (1985) argues an individual is likely to have different commitments to the goals and values of multiple groups existing within the organizational context and subsequent empirical research supports this conclusion (e.g., Becker, 1992). Further, it is noted that individuals often behave in a manner that reflects the distinctions between and among organizations and organizational constituencies (Tekleab and Chiaburu, 2011). Thus, past research has provided evidence that an organizational member is not only likely to recognize the distinctions between and among organizations and organizational constituencies, but they are also likely to have varying attitudes and change their behavior and attitudes based on these kinds of distinctions.

These studies highlight the multi-foci nature of social exchange relationships in which employees can simultaneously hold distinct perceptions toward organizations and each of the various constituencies contained within them (Reichers, 1985; Marks, 2001; Lavelle et al., 2007). As such, it appears to be plausible that employees may simultaneously perceive an exchange relationship with the organization itself and any or all of these constituencies (Marks, 2001; Conway and Briner, 2005; Lavelle et al., 2007; Alcover et al., 2017; Schuster et al., 2022a). The implication for operationalizing the psychological contract is that, rather than treating an organization as a unitary contract maker, it seems reasonable to expect that employees may simultaneously hold distinct psychological contracts with the organization itself and with each of the different agents, leading to a multi-foci perspective of the psychological contract (Marks, 2001; Conway and Briner, 2005; Schuster et al., 2022a).

There could be further complexity of contract makers in the case of expatriates, since expatriates work across MNCs and could view many agents of the wider organization as potential exchange partners (Sherman and Morley, 2016; Pate and Scullion, 2018). The recent conceptual work of Perera et al. (2018) may shed some light on the notion of multiple psychological contracts in the case of expatriates. The authors propose the idea of dual-foci psychological contracts of assigned expatriates. They maintain that in addition to the parent company, expatriates develop a psychological contract with the host company including host-company inducements of social support, language training and expatriate contributions to training and developing local co-workers (Perera et al., 2018). The authors back their argument with propositions and findings from research on expatriates’ perceptions of organizational support (POS), indicating that expatriates differentiate and hold distinct perceptions of organizational support toward the parent company and the subsidiary (Kraimer et al., 2001; Kraimer and Wayne, 2004; Liu, 2009; Liu and Ipe, 2010). In this light, the authors argue that expatriates hold a separate psychological contract with the subsidiary in addition to the one held with the parent company.

Although the conceptual work of Perera et al. (2018) has advanced the understanding of the existence of the simultaneous and distinct exchange relationships expatriates hold with the parent and host companies, it shares similar limitations with previous expatriate psychological contract studies in overlooking the potential exchange relationships between expatriates and a range of organizational agents from wider multinational corporations. However, the authors do highlight the necessity of separately identifying the party to an individual’s contract perception in further empirical examination (Perera et al., 2018). Similarly, Sherman and Morley (2016) suggest that the “senior manager in the home site, the manager of the host unit on site, and the HR director of the home unit” (Sherman and Morley, 2016, p. 17). could all be potentially perceived as the other party in an exchange relationship. Given the diverse roles they play as agents of the organization, it is important then to recognize that the “psychological contract creation process was likely to be different for each expatriate depending on the specific interaction” (Sherman and Morley, 2016, p. 17). As such, both studies suggest that in-depth examination on the foci of expatriates’ psychological contract is warranted (Sherman and Morley, 2016; Perera et al., 2018). This recognition leads to the application of the psychological contract through a multi-foci perspective, that is, employees simultaneously hold multiple psychological contracts with various organizational agents across MNCs.

In summary, this paper seeks to address some of the gaps in the literature regarding the nature of expatraites’ psychological contracts, and in particular, addresses the failure of the recent literature to consider the variation of national context and the development of concurrent psychological contracts.

## 3. Methodology

This section outlines the methodology of the study by describing the research design, the data collection and finally the approach to data analysis.

### 3.1. Research design

This paper utilizes a qualitative single-case study approach to address the research objective (Yin, 2009). The chosen setting is a state-owned automobile producer in China (Automobile Co.). Automobile Co.’s headquarters is located in Beijing; its subsidiaries are located throughout China (e.g., Chongqing Automobile Co. limited) and around the world in the United States, the United Kingdom, Italy, Japan, Mexico, Ukraine, Iran, Pakistan, and Malaysia. Initially, Automobile Co. belonged to a Chinese ordnance equipment company. But, after 14 years of integration and optimization, in 2005, Automobile Co separated from its previous owner, as an independent enterprise and now positions itself as one of the four largest automobile groups in China. In the area of automobile manufacturing, it has carried out extensive cooperation with internationally renowned automobile manufacturers (e.g., Ford and Mazda), bringing numerous classic automobile products to the Chinese market. These global investments cover its key business sectors including automobile manufacturing, components and parts, powertrain technologies and commercial services. Importantly, in the course of expanding its global networks, Automobile Co. has invested heavily in training and developing employees through exposing them to global practices and diverse technologies. As such, international assignments are often deployed to develop leadership and technical skills within the group; the duration of these assignments varies between 3 and 5 years.

### 3.2. Data collection

Data was collected *via* semi-structured interviews. This research places attention to expatriates at pre-departure stage on the basis of the likely formative nature of this episode on the development of expatriates’ psychological contract beliefs regarding their role in the upcoming international assignment (Sherman and Morley, 2016). For example, the messages conveyed from the labor market, MNCs (e.g., employment law, practice and policy, cultural value) and agents play the role of key contract-relevant information sources for expatriates to derive meanings about the reciprocal obligations within those relationships, with the created psychological contracts at this stage serving as the benchmark for understanding future employment relationships (De Hauw and De Vos, 2010). While it is acknowledged that the psychological contract beliefs are likely to be adjusted over the subsequent assignment and repatriation phase (De Hauw and De Vos, 2010; Cho et al., 2013; Sherman and Morley, 2016). For the purpose of this research, the interest lies squarely in the nature of expatriates’ psychological contracts at the start point of international assignment.

However, there was not sufficient number of pre-departure expatriates due to the case company’s own international assignment schedule. This was a situation that was beyond the researcher’s control. For the purpose of accessing expatriates’ pre-departure experiences, an adaptation of retrospective reports from the expatriates at initial entry stage was used, an approach which has proved useful in previous research into the development of psychological contract (Robinson et al., 1994; Thomas and Anderson, 1998; De Vos et al., 2003).

The participants are located in the subsidiary companies of Automobile Co. across the United States, Italy and Belgium (please refers to Table 1 for the profile of the interviewees). Given the geographical spread and location of the expatriates, interviews were conducted *via* Skype or telephone and the mode of communication was discussed with the participants prior to conducting the interviews. The interview for each participant lasts for approximately 45 min to 1 h.

**Table 1:** Profile of interviewees.

**#**	Gender	Age	Location of subsidiary company	Department in subsidiary company
1	Male	29	Detroit (United States)	Powertrain (R&D)
2	Male	40	Detroit (United States)	Transmission technology
3	Male	27	Detroit (United States)	Transmission technology
4	Male	31	Detroit (United States)	Model design
5	Male	33	Detroit (United States)	Powertrain (R&D)
6	Female	37	Turin (Italy)	Accounting
7	Male	28	Turin (Italy)	Model design
8	Male	30	Turin (Italy)	Powertrain (R&D)
9	Male	49	Ghent (Belgium)	Powertrain (R&D)
10	Female	30	Ghent (Belgium)	Model design
11	Female	27	Ghent (Belgium)	Accounting
12	Male	35	Ghent (Belgium)	Powertrain (R&D)
13	Male	47	Ghent (Belgium)	Powertrain (R&D)
14	Male	33	Ghent (Belgium)	Powertrain (R&D)

### 3.3. Data analysis

Data was analyzed through content analysis, undertaken manually. The coding frame was established “based upon a set of pre-conceived categories for which evidence is sought in the data” (Parzefall and Coyle-Shapiro, 2011, p.16). However, new or emergent codes were developed from the data and added to the initial coding framework. This was particularly utilized when the responses regarding contrac beliefs could not be accurately attributed to initial codes. Across the interviews with expatriates, the content of the psychological contracts was described and categorized according to Rousseau’s (2000) transactional, relational and balanced typology. In particular, the contract types (e.g., balanced contract) and its dimensions (e.g., performance support) respectively provided the higher-order and lower-order coding framework. This is described as follows.

*Transactional contract* is constituted by two dimensions: (1) being “narrow,” depicting the extent of involvement for both parties in an exchange relationships; (2) “short-term,” indicating the limited time duration of that exchange relationship (Dabos and Rousseau, 2004, p. 56). For example, for employment relationship categorized as transactional, employees perceived that they were obligated to “perform only a limited set of duties” and they believed their employers were obligated to offer “limited involvement in the organization, little or no training or other employee development” (Rousseau, 2000, p. 4).

*Relational contract* is comprised of two dimensions: (1) loyalty and (2) stability dimensions. For example, for employment relationship categorized as relational, employees were obligated to ‘remain with the firm, manifest loyalty and commitment to the organization’s needs and interests’ and the employers were obligated to commit to “supporting the well-being and interests of employees and their families” (Rousseau, 2000: 4).

*Balanced contract* is comprised of features of both transactional and relational contracts. Specifically, the balanced contracts are constituted by three dimensions: (1) “external employability” (developing marketable skills as employee obligations and enhancing employees’ long-term employability as employer obligations); (2) “internal advancement” (develop professional skills as employee obligations and create development opportunities as employer obligations); (3) “dynamic performance” (perform new and demanding goals as employee obligations and promote continuous learning and provide performance support as employer obligations; Rousseau, 2000, p. 5). For example, for employment relationship categorized as balanced contract, employees were obligated to “develop skills valued by both the organization and external market” and employers were obligated to “provide a range of career development opportunities and long-term stabilities in the organization” (Rousseau, 2000).

According to the theoretically-derived coding framework outlined above, the expatriates’ responses regarding the content of the psychological contract were first directly coded to into the pre-determined contract dimension categories and subsequently to the related contract type. There are instances where some expatriates’ responses across more than one contract dimension within the one statement. These were thus categorized accordingly as obligations across two dimensions. To illustrate, when expatriates were queried about the employers’ obligations, an example of expatriate response was *“the objective and timing for each stage should be carefully designed and communicated…and generally be supportive when I am unclear about something.”* In this case, this response was coded into both the performance support dimension of the balanced contract due to the focus on providing professional support, but also the loyalty dimension of the relational contract due to the focus on the employer being social–emotionally supportive when employees have personal concerns.

## 4. Findings

This section is structured to present the findings of the study by outlining the key exchange partners and the content of the concurrent psychological contracts from the perspective of expatriates.

### 4.1 Expatriates’ perceived exchange partners.

The majority of interviewed expatriates list more than one individual who are perceived as exchange partners. These include (1) headquarters-line managers; (2) headquarters-department managers; and (3) subsidiary operations.

The key exchange partners cited by expatriates are *line managers in the headquarters.* The expatriates describe their line managers as having frequent and direct involvement in their routine work. For example, the type of task, working schedule as well as the allocation of resources necessary to complete the task are all under the authority of their line managers. Several examples of the expatriates’ comments are as follows:

*My line manager must be included because most of (my) work requirements and which part (of that work) needed additional attention were determined by him.*

*I would say my line manager was quite important for my work. This was because we worked with him on a daily basis and the majority of work decisions were made by my line manager.*

Apart from being influential on how work is arranged and progressed, expatriates comment that their own performance evaluation is decisively dependent upon their line managers as well, making them the first priority in shaping the expatriates’ working experiences. As one expatriate comments:

*The performance evaluations, this was the reason why I thought my line manager was important there (the headquarters). There were quantified measures such as the prototype performance in each test run. Of course, there were also areas of quality control where he would like to see more effort from me… mostly he was the one taking charge of these things.*

Expatriates also cite their *department managers* as critical exchange partners. While admitting that the interactions with their department managers in the workplace were not frequent in comparison to their line managers, the expatriates recognize their higher hierarchical position and significant influences in relation to the design and facilitation of organization policy and practice. An example of the expatriates’ comments is illustrated as follows:

*The department manager was pretty important as well. He often set and communicated objectives when there was a new quality standard adopted to evaluate our product or initiatives on hybrid power development.*

Furthermore, some crucial work-specific decision-making is made by the department managers, as one expatriate comments:

*He (department manager) often participated in some major work-related decisions like deciding the fundamental models for simulation. I also sought advice from him in some critical parameters in this regard.*

The authority of department managers in making personnel appointments is also the focus of the expatriates’ comments. There is a general agreement among these expatriates that although there are standard organizational policies in the area of promotion, these are only considered guidelines. In practice, the department managers hold a determinant role in the decision of promotion opportunities. As one expatriate comments:

*I don’t consider the system of promotion clear, and it (promotion policy) does not work like that. The policies stated that if you were performing above average in terms of appraisal categories, then you were eligible for a promotion, but it did not say that you will be promoted. If you thought it over, it was up to your department to nominate you, so basically your department managers were the decision-makers, and it was actually the case.*

Many expatriates also identify the *subsidiary unit* itself as an exchange partner due to its potential influences on their upcoming work. It is noteworthy that expatriates find it difficult to specifically outline agents or departments of subsidiary units as influential, with some explaining that without sufficient knowledge of the subsidiary, they assume that the number of people who would provide support or make decisions influencing their work progression would be quite large. This is illustrated by the following comment:

*I guess it depended on a lot of people, if your project needs finance, you need approval from the finance department… if you need an extra hand, you need your department managers or HR; and maybe you were not aware of if someone helped you.*

The following set of findings present the results on the content of the concurrent psychological contracts. This is structured according to (1) expatriates’ perceptions on the obligations of the exchange partners (employers’ obligations thereafter); and (2) expatriates’ perceptions regarding what they should provide in return to the exchange partners (employees’ obligations thereafter). Please refer to Table 2 for details.

**Table 2:** The content of expatriates’ psychological contracts.

Theme	Theme description	Exchange partners	Example quotes
**Employers’ obligations**			
Performance support (balanced contract)	Employees’ perceptions on the obligations of employers in terms of providing work performance-related support.	Headquarters-line managers	*“He (my line manager) was also specialised in engineering, so his participation in the technological part of the work made my project run more smoothly… I mean, you will have an idea of which direction to go.”*
		Subsidiary units	*“Creating a people-oriented, friendly and supportive environment; a place where you know where you could get help.”*
Development (balanced contract)	Employees’ perceptions regarding the obligations of employers in terms of promoting continuous learning and development opportunities	Headquarters-department managers	*“I do appreciate if there are opportunities to work on a project of different nature to expand my skills and knowledge.”*
		Subsidiary units	*“I thought that they should invest more in programmes or practices that will allow my self-development.”*
Loyalty (relational contract)	Employees’ perceptions regarding the obligations of employers in terms of supporting the well-being and interests of employees and their families.	Subsidiary units	*“I think you would like to know their policy about employee welfare, this shows how much this company values the interest of its employees.”*
**Employees’ obligations**			
Performance support (balanced contract)	Employees’ perceptions regarding what they are obligated to provide in return to employers in terms of meeting new and demanding goals.	Headquarters-line managers	*“I thought it was to fulfil your duties properly in the assignment… to fulfil what was expected of you… and to devote your efforts to the goals of this team.”*
		Subsidiary units	*“I thought they will need employees who were efficient and committed. So, my obligations may include… improving my capabilities… and we also need good teamwork because we will work with people from diverse cultural and educational backgrounds.”*
Development (balanced contract)	Employees’ perceptions regarding what they are obligated to provide in return to employers in terms of developing professional skills.	Headquarters-department managers	*“I believed what my department managers expected should be developing myself and using this assignment as an opportunity to learn and apply my skills.”*
		Subsidiary units	*“It should be learning as fast as we can. As I mentioned before, we (headquarters and the subsidiary) certainly had gaps in terms of technical strength, so we need to learn quickly. This will be an opportunity for self-development and certainty will be an advantage when we go back (to their home country).”*

### 4.2. Employers’ obligations

The expatriates are quite clear in articulating their respective perceptions regarding the obligations of their exchange partners, where a few key beliefs emerge and are categorized utilizing Rousseau’s (2000) contract typology. Overall, employers’ obligations focus predominately on the performance support dimension and development dimension of the balanced psychological contract, as well as the loyalty dimension of relational contract.

The employers’ obligations on performance support relate to both of headquarter-line managers and subsidiary unit itself. In describing these beliefs, expatriates clearly give priority to the importance of open and direct communication with their respective line managers. Overall, the issue of communication was raised in all 14 of the interviews and brought up more than once in each of the interviews. Specifically, expatriates felt a pressing need for clear guidance on the precise nature, timing and objective regarding the forthcoming expatriation assignment. This is illustrated by the following comments:

*I did expect him (line manager) to be clearer about the project I will be doing… the objective and timing for each stage should be carefully designed and communicated.*

*I think there should be more feedback on how the project will proceed, especially when there were conflicts of opinions on some critical input data (of simulation).*

There is also an expected communication from line managers with regard to the relational aspects of subsidiary operations. For example, the operating style and personality of managers in the subsidiary units, preparing these expatriates for networking in a new environment. As one pre-expatriate comments:

*I was more interested in the people… like my future managers there; how does he prefer to handle things, how to develop work relationships there… and who should I go to if I need help.*

Furthermore, the performance-related support extends to expatriates expecting role-specific support, such as consulting on technical details of tasks. This is illustrated by the following comment:

*I expected especially more instruction on this job from my line manager, this let me know whether I am on the right track, how I am doing and whether there is something to be improved.*

In terms of subsidiary units, the performance support-related beliefs are generally referred to as a supportive environment. This is likely due to the expatriates being uncertain concerning the source of their future support, therefore tending to aggregate these beliefs toward potentially varied organizational agents within the subsidiary units at an organizational level. As one expatriate states:

*Before this assignment, the general expectation was about support. But it was not very clear at that time what kind of support it will be, so, the most important thing for me should be a supportive environment.*

The employers’ obligations on development relate to headquarter-department managers and subsidiary unit itself. The key beliefs clustered under this category relate to the provision of opportunities to develop job skills valued by the organization as well as by the external market (e.g., qualification certificates), typically through the provision of a structured training program. For example, several expatriates comment:

*I think (my department manager’s obligation) was more about providing training, like the qualification I am working on.*

*This was mostly about developing my job skills on engine control technology, because my department manager was an expert in this area, I believed he should have an idea about the future development of this professional area.*

Furthermore, expatriates seek a path of career advancement and such beliefs are deemed to be the responsibility of their department managers who are perceived to be influential in making personnel appointments. Expatriates’ comments suggest that the experience of undertaking an international assignment would differentiate them from domestic employees and would be rewarded with high-level job prospects upon repatriation. Several examples of the expatriates’ comments are as follows:

*Before I went on an international assignment, I thought these experiences would mean that I could develop my career more quickly than others.*

*There were certainly opportunities to learn from the complexity of the tasks and the experience of coordinating with other professionals.*

*I thought the international experience would give me something valuable that differentiated me from my peers, at least (the expatriation) will give me more opportunities in the company than before.*

The responses in relation to subsidiary units tend to be overlapped to some extent with the results found for headquarters department managers, such as opportunities for upskilling and career progression. For example, one expatriate comments:

*I considered this assignment as an opportunity to further my career, so it would be expected that they had opportunities to challenge myself.*

The employers’ obligations regarding the loyalty dimension of the relational contract relate mainly to subsidiary unit. These beliefs focus largely on social–emotional support through creating a friendly environment. For example, an expatriate comment:

*At the pre-departure stage, it should be concerned with more support in my life.*

### 4.3. Employees’ obligations

While the concept of the psychological contract is rooted in the notion of reciprocal exchange, much of the empirical work on the content of contracts focuses solely on the employees’ perceptions of employer obligations, reflecting a scarcity of studies that assess both perceived employer and employee obligations when operationalizing the psychological contract. Responding to this point, this paper seeks to assess perceived employers’ obligations while exploring employees’ perceptions regarding what they should provide in return in exchange relationships.

Overall, the employees’ obligations focus predominately on the performance support dimension and the development dimension of the balanced psychological contract. The expatriates generally sent consistent messages in articulating their performance support-related obligations towards headquarter-line managers and subsidiary unit itself. These include properly fulfilling their responsibilities in the assignment, achieving the designed objectives, working with efficiency and being a team-player. Several examples of expatriates’ comments are illustrated below:

*I would say, first, to achieve the performance standards you were required at first.*

*It was simple for me: to perform your work effectively and generally work hard.*

*Being active in working with their teams and try to take on more obligations.*

The employees’ obligations on development toward headquarters-department managers and subsidiary unit also largely overlap with each other. These include, for example, expansion in professional knowledge, a willingness to learn and actively seeking development opportunities. It is highlighted that some expatriates find it difficult to distinguish the obligations of the subsidiary from those of their headquarters-department managers. For example, several expatriates comment:

*I guess… he (the headquarters department manager) thought that I should have an active attitude and be willing to learn.*

*Before going abroad, it (my obligation) was largely about performing our work according to the outlined plan… I thought it was the same for subsidiaries; what I can offer will be my work performance.*

## 5. Discussion

The objectives of this study are to identify the key exchange partners with whom pre-departure expatriates create psychological contracts [research question 1(a)] and on the basis of this, to specify the content of perceived employer as well as employee obligations [research question 1 and 1(b)]. The following sections now turn to the discussion of the results obtained in these two areas.

### 5.1. Exchange partners from a multi-foci perspective

The results in this regard confirm and extend the psychological contract literature and the constructs rooted in social exchange theory in general in terms of the understanding of the parties to employee-organization relationships (EOR). A wide variety of organizational behavior theories drawing on the idea of social exchange have extensively examined different forms of exchange relationships that are operationalized through the parties involved in the relationships. For example, leader-member exchange (LMX) captures the quality of relationships formed with immediate managers (Kunze and Phillips, 2012); team-member exchange (TMX) represents an individual quality of relationship with co-workers (Shore et al., 2004). Meanwhile, perceived organizational support (POS) offers an indication regarding employees’ perceptions of resources and support actually received from the organization (Guchait et al., 2015). From this lens, the findings regarding pre-departure expatriates’ perceived exchange partners confirm what other authors have found to be the salient parties for exchange relationships in work settings.

Further to this, while psychological contract resides within an individual as perceptions of reciprocal obligations with another party, constructs including LMX, TMX and POS depict the ‘perceptions of actual state of relationships’ with the immediate leader, co-workers and organization itself, respectively, (Shore et al., 2004). Although it is not the intention of this research to differentiate among the outlined constructs (please see Shore et al., 2004, for details), it is important to note that the assumptions underlying these constructs are a series of interdependent interactions where one party’s behavior is contingent on the other party (Shore et al., 2004; Cropanzano and Mitchell, 2005). In relation to psychological contracts, a strict interpretation of social exchange theory would lead to both the inducements (perceived employer’s obligations) and contributions (perceived employee’s obligations) being defined in terms of a single partner. The results of this study suggest a more nuanced view of the psychological contract in that reciprocal obligations tend to be defined in terms of multiple foci.

To clarify, the most frequently listed exchange partners by the pre-departure expatriates are line managers and department managers in the headquarters. Importantly, individuals appreciate the respective role of each party in shaping their aspects of work conditions while acknowledging the simultaneous existence of such influences. Specifically, it is found that expatriates often have direct and the most contact with their line managers who often play a role in work-specific issues such as their type of task, workload, the allocation of resources and performance evaluation. Relatedly, the department managers (middle-level managers) are typically perceived as influential decision-makers in terms of facilitating organizational policy/practices and personnel appointments. Despite the debate on the issue of employer representation (the type of agent who is in the best position to capture the view of the organization), the results obtained here are consistent with the psychological contract literature in the sense that first, “most contract makers are individuals acting as the organization’s agents” (Rousseau, 1995, p. 60), and second, employees’ line managers (Lewis and Taylor, 2001; Tekleab and Taylor, 2003) and middle-level managers (Coyle-Shapiro and Kessler, 2000) are both theoretically and empirically positioned to be the key contract makers to an employee.

To extend the discussion on contract makers, the implication of the subsidiary itself acting as another party to expatriates’ psychological contracts is discussed below. While the agents of organizations offer more specific guidance on employment relationships, literature looking at how the psychological contract state (e.g., contract violation and fulfillment) influences employees’ outcomes tends to adopt a view that the organization as a whole is assumed to be the other party in the psychological contract of an employee (Lee et al., 2014; Ruiter et al., 2018). This is particularly the case for research using surveys as data collection tools (Chi and Chen, 2007; Chen and Chiu, 2009; Chen, 2010). These studies rest on the assumption that rather than a specific agent, individuals view all possible contract makers including agents and functional or administrative departments bundled into a single exchange party. With reference to the psychological contract theory and EOR in general, this process is referred to as “personifying” or “anthropomorphizing” where employees can “view all the organization’s possible agents, principals, and non-human contract makers (e.g., departments) as if the organization were a single, human, contract maker” (Conway and Briner, 2005, p. 128).

Anthropomorphising of organizations can be traced back to Levinson (1965) whose position, as summarized by Conway and Briner (2005, p. 130), is that “employees view actions by agents of organization as actions by the organization itself.” This process is facilitated by organizations’ legal, moral and financial responsibilities regarding the actions of agents and as a result, the “organization takes on an anthropomorphic identity as a party to the psychological contract” (Robinson and Morrison, 1995, p. 290). Such a process bears a close resemblance to the observation made within this study with the identification of the subsidiary operation itself as an exchange partner. Specifically, individuals show an intention to view all potential contract makers (organizational agents and departments) in subsidiaries as the sources of support provided by the subsidiary operations, therefore implicitly treating the subsidiary unit as a single exchange partner. This is also evident by expatriates using the terms ‘they’ or ‘the company’ to relate to a subsidiary when describing the associated contract beliefs.

### 5.2. The content of the psychological contract from a multifoci perspective

The findings for research extend the understanding of expatriates’ psychological contracts by qualitatively exploring the foci-specific content of pre-departure expatriates’ psychological contracts. The content of these contracts comprises the individual’s understanding of the reciprocal obligations between the focal individual and another party and this is categorized utilizing Rousseau’s (2000) contract typology.

Existing contract research works from the viewpoint that each employee establishes one psychological contract with a unitary “employer” that is represented either by a unique person (immediate manager, HR unit, or senior manager) or organization in itself (Sherman and Morley, 2016; Haak-Saheem et al., 2021; Koveshnikov et al., 2022). While studies has offered evidence that employees differentiate the level of social exchange they perceive themselves having with multiple parties, and there have been theoretical propositions about the multi-foci perspective of the psychological contract (Marks, 2001; Lavelle et al., 2007), limited research has been done to explore these issues.

#### 5.2.1. Employers’ obligations

Drawing on the multi-foci perspective in exploring reciprocal psychological contract beliefs, the pre-departure expatriates’ psychological contracts can be categorized as predominantly balanced contracts, with low-level dimensions of performance support and development being found to vary to some extent according to the foci of that exchange relationship.

Zoom in on these themes offers a more nuanced understanding of Chinese expatrates’ psychological contracts. The expatriates’ beliefs regarding the performance support dimension relate mainly to headquarters line managers while those regarding development dimensions relate to headquarters department managers. In looking at the contract beliefs specifically at the item-level, while individuals attribute obligations such as open communication, regular feedback and technical support to the responsibilities of line managers, beliefs including opportunities of developing valuable job skills and career prospects are perceived as the obligations of department managers. Moreover, although the expatriates’ beliefs regarding subsidiary units cross both dimensions of the balanced contract (performance support and development), it is important to note that these tend to be differentiated from the beliefs regarding the other two contract makers at the contract-item level (e.g., supportive environment).

Drawing on the proposition by Coyle-Shapiro and Parzefall (2008) may offer a plausible explanation for the distinct beliefs of each contract maker. It was suggested that the role of immediate and middle/senior managers may be complementary in managing employee-organization relationships. Employees may develop multiple relationships: “a distal relationship with senior managers and a proximal one with line managers” (Coyle-Shapiro and Parzefall, 2008, p. 20). Corresponding to this notion, the results obtained here illustrated that department managers are the key decision-makers in defining the broad parameters of the exchange (design and facilitation of organizational practice/policy and personnel appointment), therefore being attributed with associated obligations, typically training practice and career progression. Meanwhile, managers in the lower level of organizational hierarchy (line managers) enact these policies on a day-to-day basis and therefore define the specific elements of the exchange (e.g., type of work and resource allocation), thus further being associated with obligations related to those matters (e.g., communication and technical support). This suggests that, as a function of authority, the exchange partners differing in the level of organizational hierarchy appear to be responsible for maintaining different aspects of work conditions, potentially adding more details and specificity to the establishment of expatriates’ foci-specific psychological contracts.

#### 5.2.2. Employees’ obligations

In relation to employees’ perceived obligations, two points are made. First, the differentiation of contract dimensions is also evident for employees’ obligations: expatriates’ obligations regarding the performance dimension (e.g., work with efficiency) is related mainly to their line managers whereas those regarding the development dimensions (e.g., expansion in professional knowledge) is related to their department managers.

Second, while the results for employers’ obligations regarding the upcoming international assignments tend to be specific with expatriates attaching more detailed descriptions, this is the opposite for employees’ perceived obligations. Indeed, they are general and non-context-specific. For example, expatriates often refer to their domestic working experiences (e.g., the nature of work and reporting procedures) in articulating the performance support-related responsibilities of headquarters line managers, whereas the corresponding employee obligations are quite broad and generic, focusing largely on providing high-quality work performance. A plausible explanation could be derived by looking at the nature of contract beliefs constituting the psychological contract. Psychological contracts are belief systems of individual employees and employers regarding their mutual obligations; the development of these contract beliefs depends on the promises made as employment arrangements (Hui et al., 2004). These promises can be conveyed through words (explicit promises) or actions (implicit promises; Rousseau, 1989).

In both cases, the context in which the employment relationship is embedded plays a key role in establishing whether a promise has been made (Rousseau, 2001). For example, organizational communications, whether in their explicit or implicit forms, are more likely to be interpreted by individuals as promises in the events in which repeated interactions occur (e.g., recruitment and socialization: Rousseau, 2001). Returning to the case of expatriates, the specificity of perceived employer obligations for the line managers and department managers is likely due to the existing employment relationships offering the context in which the repeated interactions occurred. For example, expatriates often describe the direct and frequent involvement of line managers in directing their routine work. Thus, when verbal expressions and actions occur on such occasions, these are more likely to be interpreted as promises that are further used to construct psychological contract beliefs (Rousseau, 2001). In contrast, this is not the case for perceived employee obligations given the event of international assignment has not commenced yet from the perspective of pre-departure expatriates, hence the relative absence of promises. From a methodological lens, the inherent limitations in the data collection methods (the adaptation of retrospective reports) may also result in the non-context-specific nature of perceived employee obligations. However, this seems to be unlikely given the specificity of employers’ obligations.

Overall, the results based on the Chinese expatriates in this sample contrast to what the other authors find to be salient beliefs for Western expatriates, where the overall tone is transactional (Pate and Scullion, 2010; McNulty et al., 2013). The differences in the content of expatriates’ psychological contracts could be attributed to cultural values. In general, expatriates sent from the Western context (e.g., United States) tend to be classified as individualism with some regional variations (Vandello and Cohen, 1999). As observed in the study of Pate and Scullion (2010, p. 68), the transactional arrangement between expatriates and employers were characterized by expatriates being “less reliant on any one organization” and “negotiating their contract more aggressively.” These appear to be in line with the key features of individualism such as autonomy and independence (Singelis et al., 1995).

In contrast, Chinese culture is based on Confucianism and Taosim. This value emphasizes hierarchy and harmony, which are characteristics of collectivisim orientation (Thomas et al., 2010; Du and Vantilborgh, 2020; Yao et al., 2020). It was clear that Chinese interviewees felt a strong obligation to obey their superiors. This is evident in that they accepted a greater management control over working procedures; and they legitimized the differences between line managers and department managers on the basis of hierarchical positions. Likewise, they believed that the employers were obligated to offer clear work instructions to follow, while to act as the decision-makers when conflicts of opinions arose. These beliefs fit with a strong deference to authority. In addition, Chinese interviewees value harmony, which translated in to the psychological contract beliefs such as being team player. Of note, this paper acknowledges the inconsistency with the findings based on national employees in Hong Kong. This is also where the majority of the psychological contract research into China has concentrated (e.g., Kickul et al., 2004; Zhao et al., 2007; Ravlin et al., 2012). The employees in Hong Kong, in general, were reported to have a transactional psychological contract. The key contract beliefs focused on employers’ obligations to provide economic benefits (Kickul et al., 2004), albit that Hong Kong and mainland China share a common culture value of Confucianism. While it has been acknowledged that expatriates’ psychological contracts are different from the psychological contracts of national employees (Zhang and Rienties, 2017; Ruiter et al., 2018), it is possible that the employees in Hong Kong have been influenced more by a Western culture than their counterparts in mainland China (Du and Vantilborgh, 2020). The implication being that the focus on South China (Hong Kong) may provide a biased view of Chinese culture heritage. Thus, when exploring the psychological contract of Chinese employees, the broader psychological contract literature, and the expatriates’ psychological contract research in particular, may benefit more from accounting for the regional variation including the North and Central China.

## 6. Implications

The objective of this research is to examine the content of Chinese expatriates’ psychological contracts from a multi-foci perspective. Overall, the findings offer evidence that the expatriates in this research have multiple psychological contracts simultaneously, each with a different foci. The presence of multiple parties differing in the level of organizational hierarchy add additional details to an individual’s foci-specific psychological contract. This is evident in terms of the varied contract dimensions and the reciprocal obligations contained. Further, the Chinese expatriates in the sample have predominately balanced contract beliefs, which contrast sharply to what the other authors find to be salient beliefs (e.g., transactional contract beliefs) for expatriates based on Western samples.

This paper contributes to the psychological contract literature in two main respects. First, it provides a clearer description of with whom an individual has a psychological contract. There is a growing body of evidence demonstrating that multi-foci perspectives are more precise than single-foci perspectives when attempting to predict the attitudes and behaviors of organizational members (e.g., Reichers, 1985; Shore et al., 2004; Lavelle et al., 2007; Cropanzano and Rupp, 2008). Thus, it is important to understand the degree to which individuals distinguish between exchange partners. This helps to ensure that researchers do not consider redundant relationships. With particular attention to the psychological contract literature, researchers can be encouraged to draw finer distinction among multiple exchange partners, rather than assuming the employment relationship is adequately captured by an overarching construct.

Second, this study also contributes to the discussion of the nature of expatriate psychological contracts. The employment relationship of expatriates being balanced in character diverges from the standpoints in prior research (e.g., Pate and Scullion, 2010; McNulty et al., 2013). Therefore, it is possible to unpack aspects of national cultural values. This may shape the way in which the reciprocal obligations that constitute the psychological contract are formed. It is suggested that further work in this area is required.

The findings also offer a range of insights for managers and other organizational representatives regarding expatriate management. It is clear that expatriates maintain differentiated exchange relationships with organizational members of the wider MNCs. Within these relationships, the expatriates are able to distinguish among a varied range of organizational agents and clearly, a varied importance are placed upon them by the expatriates. In this case, managers are encouraged to understand which exchange relationships are the most important to expatriates and to identify the content of these important psychological contracts. This helps to enhance management’s understanding on the motives and demands of those expatriates. In addition, expatriates use the perceptions for their line managers and middle-level managers as the basis for an overall assessment on what they believe they should be giving and receiving in their employment relationships. As the potential organizational agents can be variable, fostering these types of relationships can be an effective strategy for organizations to encourage expatriates’ retention.

## Data availability statement

The datasets presented in this study can be found in online repositories. The names of the repository/repositories and accession number(s) can be found at: https://pan.baidu.com/s/1cGLJI_AaT1gJfAOUkZbuRg.

## Ethics statement

The studies involving human participants were reviewed and approved by Ethics Committee of University of Glasgow. The patients/participants provided their written informed consent to participate in this study.

## Author contributions

XW, SW, and MM contributed equally to the design of the article, revised it critically for important intellectual content, approved the version to be published and agreed to be accountable for all aspects of the work. All authors contributed to the article and approved the submitted version.

## Conflict of interest

The authors declare that the research was conducted in the absence of any commercial or financial relationships that could be construed as a potential conflict of interest.

## Publisher’s note

All claims expressed in this article are solely those of the authors and do not necessarily represent those of their affiliated organizations, or those of the publisher, the editors and the reviewers. Any product that may be evaluated in this article, or claim that may be made by its manufacturer, is not guaranteed or endorsed by the publisher.
